# Penile Microdissection: A Live Donor Feasibility Study in Feminizing Gender-Affirming Surgery

**DOI:** 10.3390/life13112212

**Published:** 2023-11-15

**Authors:** Slavica Pusica, Borko Stojanovic, Marko Bencic, Marta Bizic, Tatjana Atanasijevic, Miroslav L. Djordjevic

**Affiliations:** 1 Belgrade Centre for Genitourinary Reconstructive Surgery, 11000 Belgrade, Serbia; slavica9212@gmail.com (S.P.); stojanovic@uromiros.com (B.S.); bencic@uromiros.com (M.B.); martabizic@uromiros.com (M.B.); 2 School of Medicine, University of Belgrade, 11000 Belgrade, Serbia; gatanasijevic@sbb.rs

**Keywords:** penis, anatomy, microdissection, gender-affirming surgery, transplantation

## Abstract

Femininizing gender affirmation surgery includes the creation of external female genitalia such as a new clitoris, labia, and vagina with removal of the glans and urethral remnants and full corpora cavernosa. We evaluated the possibility of using preserved cavernosal bodies with glans and urethral remnants for potential live-donor penile transplantation. Between March 2021 and February 2023, penile microvascular dissection followed by gender-affirming vaginoplasty was performed in 41 patients aged 18 to 57 years (mean 30.5 years). The mean follow-up was 15 months (ranging from 6 to 26 months). The removed penile entities were properly measured. The corpora cavernosa were completely preserved in all cases; the length of remaining anterior urethra ranged from 12.70 cm to 16.40 cm, while the mean glans remnant volume was 85.37% of the total volume. All patients reported satisfactory results after gender-affirming vaginoplasty. Microvascular penile dissection in gender-affirming vaginoplasty is simple and safe, suggesting a good possibility of using the full corpora cavernosa, glans, and anterior urethra remnants for live-donor penile transplantation.

## 1. Introduction

Penile transplantation presents an ideal method for the treatment of penile agenesis, penile hypoplasia, and loss of the penis after trauma or penile cancer. Additionally, it could be an optimal option for transmen who seek male genitalia. Penile deficiency in all these conditions can lead to severe psychological and psychosexual impairment. Phalloplasty still remains the only option for penile restoration in all these indications. Despite a variety of surgical techniques and tissue transfers that have been reported for phalloplasty so far, none present an ideal option [1,2,3]. Multistage surgery, high complication rate and poor results in erectile and voiding functions in total phalloplasty induced ideas about possible penile transplantation. In the last two decades, just five allogenic human penile transplantations were performed in cis men from cadavers. The first penile transplant was performed in Guangzhou in 2006, resulting in explantation two weeks later due to the patient’s discomfort with the penile appearance and deformities [4,5]. Two cases in Cape Town were followed from 2014, with good erectile function, but the first one complicated with several sexually transmitted infections, urethral strictures, and partial graft necrosis [6]. Another one (2017) developed rejection episodes two years later with pain and necrosis leading to explantation in 2021 [7]. Both US (Boston and Baltimore) cases had intermittent episodes of acute rejection but have been stable and remain under continual monitoring [8,9].

On the other hand, feminizing gender-affirming surgery presents the last step in the transfemale surgical transition. It includes a bilateral orchidectomy, penectomy, clitoroplasty, labioplasty, and vaginoplasty. Penile inversion vaginoplasty still presents the gold standard technique, while intestinal and peritoneal pull-through vaginoplasties are usually reserved for re-do cases and cases with insufficient penile skin [10,11,12,13]. The new female genitalia are created from the penile and scrotal skin, a piece of the glans with neurovascular elements, and the proximal part of the urethra. All other parts, such as the completely preserved corpora cavernosa, a large volume of the glans remnant, and the distal penile urethra, are unnecessary and are usually removed from the body. We theorized that these parts of the penis could be used as tissue for live-donor penile transplantation. Dissection of the penile entities presents a base for the construction of the new female genitalia: penile and scrotal skin is used for the vaginoplasty and labioplasty, a small part of the glans with the neurovascular bundle is used for the clitoroplasty, and a proximal part of the urethra is used for the neovaginal vestibulum and female urethral orifice [14]. We have evaluated possibilities for the preservation of all remaining penile structures during femininizing gender-affirming surgery as a potential tissue for live-donor penile transplantation. Usually, corpora cavernosa with a glans remnant after clitoroplasty and distal urethra after vulvoplasty, are removed. We hypothesized that completely preserved cavernosal bodies with a good volume of remaining glans tissue and anterior urethra present viable tissue to be used for potential live-donor penile transplantation.

## 2. Materials and Methods

Between March 2021 and November 2022, we performed feminizing gender-affirming surgery on 41 transfemales, aged from 19 to 57 years (mean 30.5 years). The surgery was performed after a minimum of one year under hormonal therapy in all cases. There were no cases of congenital anomalies of the genitalia in this cohort. Surgery included anatomical dissection of male genitalia, followed by reconstruction of new female external genitalia. In all candidates, the penis consists of a non-deformed corpora cavernosa, well-developed skin without scars, and good volume of the glans and urethra without any deformities. All penile structures were separated without complications and prepared for the creation of the female genitalia according to standard feminizing gender-affirming surgery. Penile inversion and peritoneal pull-through vaginoplasty were performed in 27 and 10 cases, respectively. In the remaining 4, sigmoid vaginoplasty was performed due to a lack of available skin after radical circumcision in childhood. This study was presented to the University of Belgrade as a part of the IDEAS 2020 project and approved by the Ethics Committee (No. 1322/X-16). Informed consent was obtained in each case, and all information about the patients was confidential and protected according to ethical principles.

**Figure 1:**
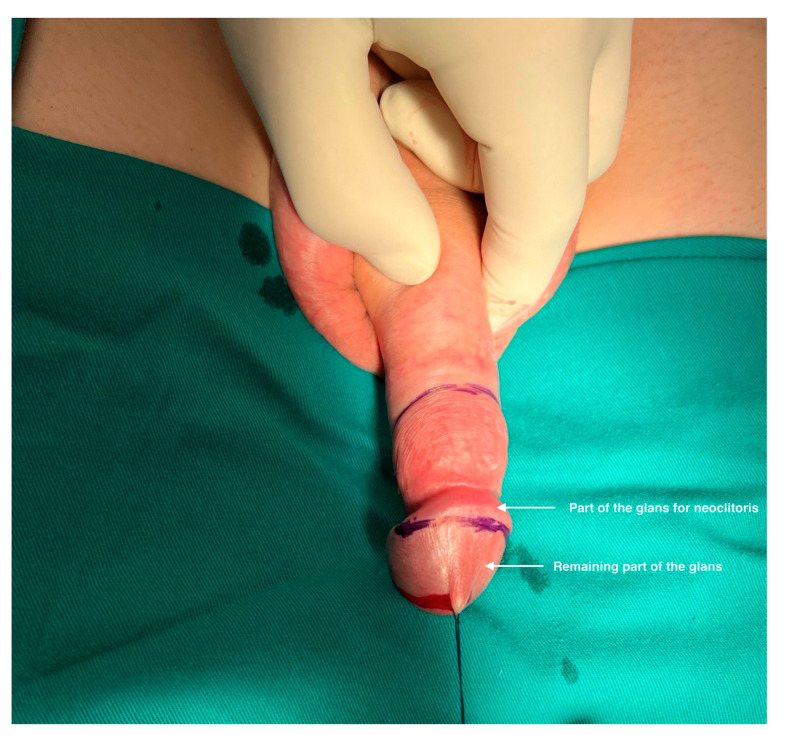
Appearance of male genitalia before feminizing gender-affirming surgery. Part of the glans and prepuce is marked for incision.

**Figure 2:**
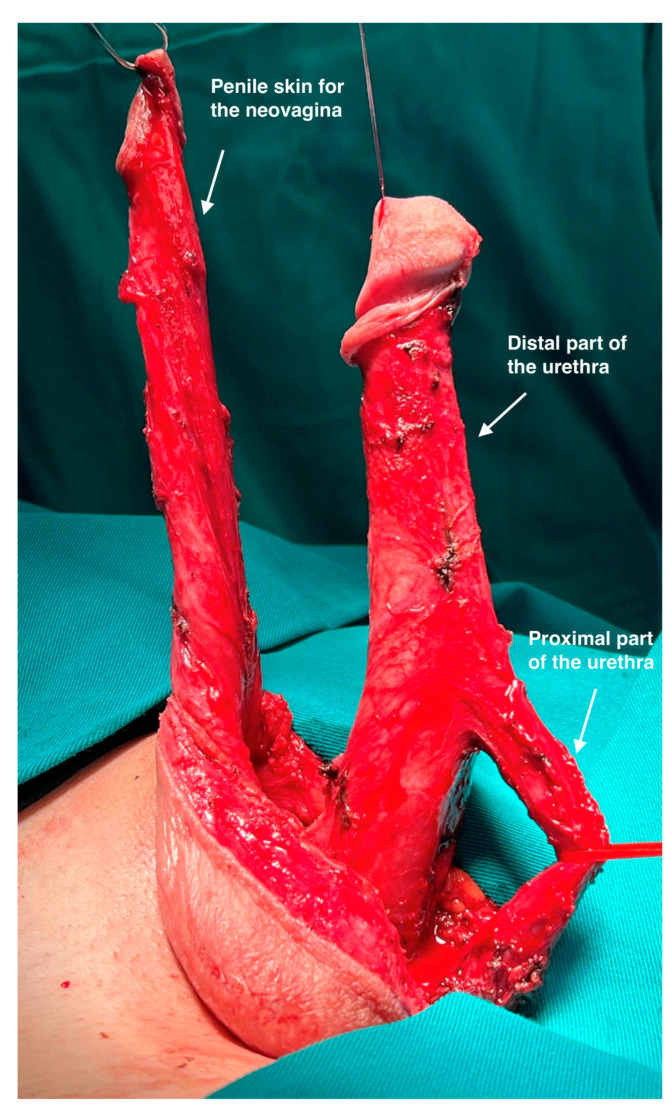
Degloving is performed and penile skin is completely preserved for vaginoplasty. Ventrally, bulbar urethra is mobilized and prepared for division. Distal part of the urethra is attached to the corpora cavernosa.

Macro-dissection was performed in a standard fashion and revealed the usual penile anatomy without penile deformities in all cases. (Figure 1) The approach was based on our vast experience in penile disassembly, which was introduced for the treatment of congenital and acquired penile anomalies [15,16]. Penile entities are the focus in this phase, and meticulous micro-dissection is performed. A circumferential incision is performed 3 cm below the glans corona allowing degloving of the penile skin from the corpora cavernosa and urethra, using sharp and blunt dissection. An additional longitudinal incision along the scrotal raphe is made for penile shaft inversion and subsequent bilateral orchidectomy. The penis is then invaginated through the incision to provide excellent access through its entire length. Dissection of the urethra starts laterally in Buck’s fascia layer with two linear paraurethral incisions on each side of the urethra. Proximally, the dissection comprises the bulbar urethra that is divided. In this way, the proximal part of the urethra is prepared for the reconstruction of the vestibulo-vaginal complex and the new female urethral orifice. The anterior urethra remains attached to the corpora cavernosa, preventing injury of the connection blood vessels (Figure 2).

**Figure 3:**
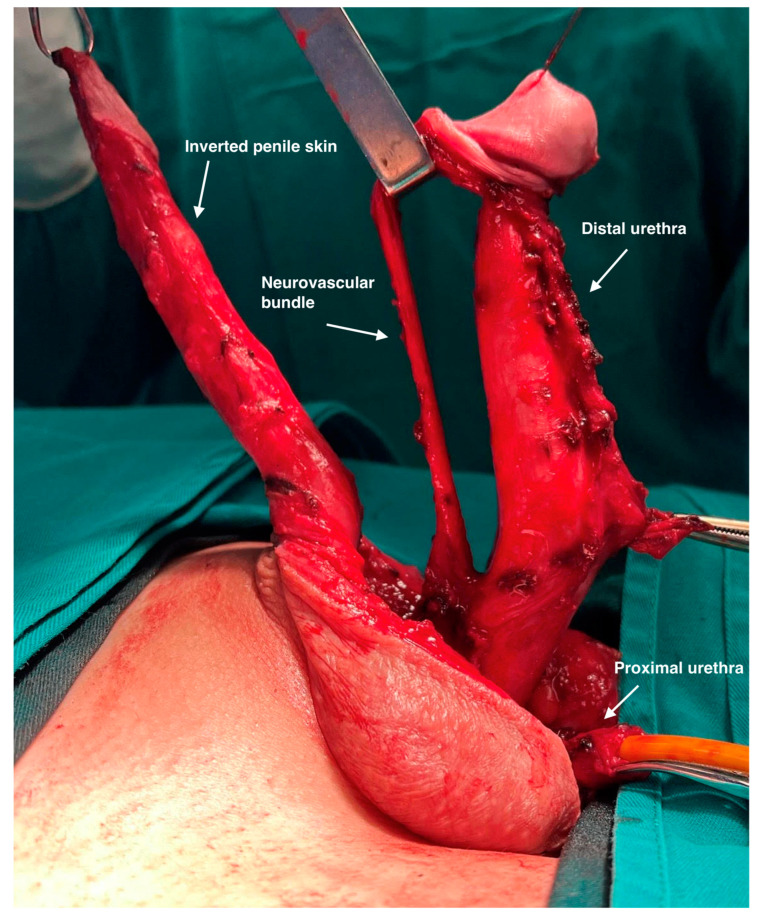
Dorsally, the neurovascular bundle is lifted from the corpora cavernosa. Partial dissection of the dorsal part of the glans is performed. Ventrally, urethra is divided, and distal part remains attached to the corpora.

The neurovascular bundle evaluation includes neurovascular structures such as the deep dorsal vein, two penile arteries, and two penile nerves. Neurovascular elements are very precisely lifted from the tunica albuginea using combined sharp and blunt dissection in Buck’s fascia layer. The deep dorsal vein can be seen running longitudinally in the middle of the neurovascular bundle, surrounded by one dorsal artery and one dorsal nerve on each side. Distally, the dissection is continued to the dorsal surface of the glans. (Figure 3).

**Figure 4:**
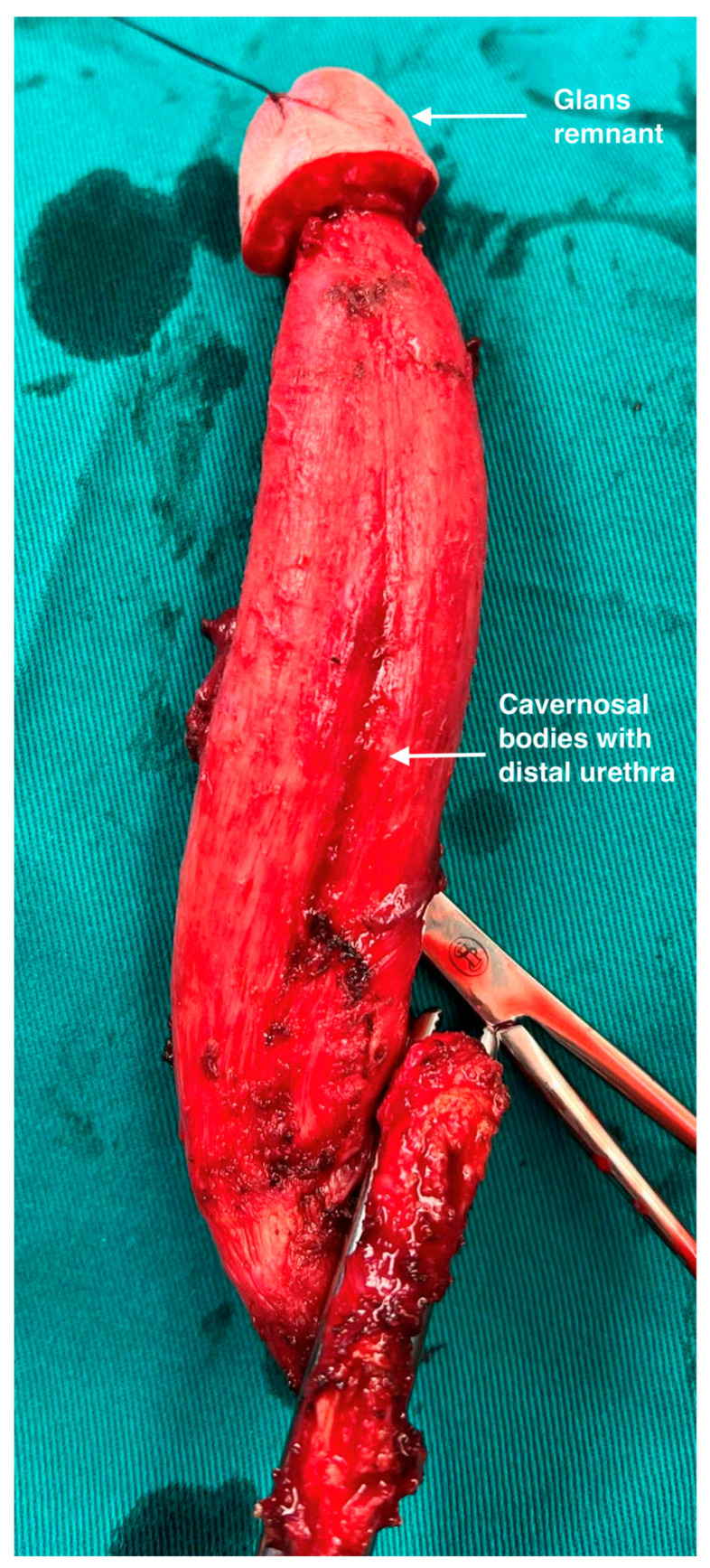
Completely preserved corpora cavernosa are removed. Good volume of the glans remnant as well as distal urethra are attached to the corpora cavernosa.

Part of the glans is lifted to a level determined by the appropriate size of the new clitoris. The approximately 1 cm wide coronal ridge is divided from the glans and coned to shape the new clitoris. The remaining part of the glans is left in place, attached to the tips of the corpora cavernosa. Resection of the crura of the corpora cavernosa is performed with its ligation at the level of the pubic bones. This way, complete cavernosal bodies with part of the anterior urethra and glans cap are removed (Figure 4).

**Figure 5:**
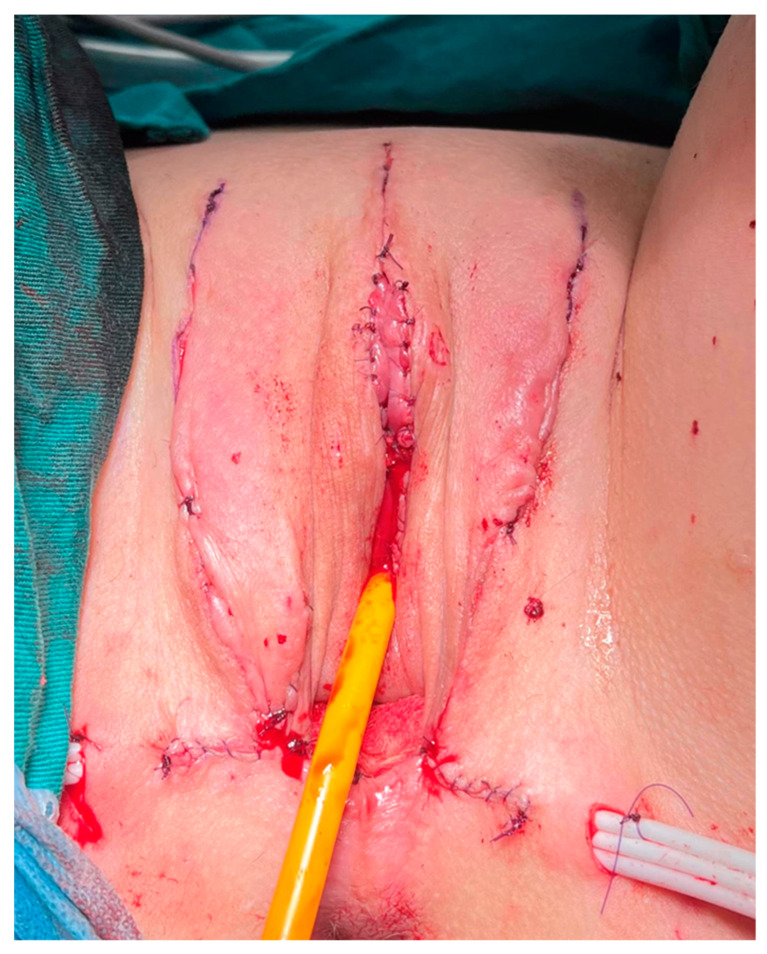
Final appearance after gender-affirming surgery. Good esthetical appearance is achieved.

Finally, the penile skin is inverted and used to create the vaginal introitus and distal part of the neovagina. Additional tissues, either scrotal skin grafts, peritoneal flaps, or the sigmoid colon flap, are joined with the inverted penile skin, giving full depth of the neovagina. The remaining genital skin is used for labioplasty (Figure 5).

Our dissection included precise measurement of the following penile elements: length and girth of the corpora cavernosa after removal, length of the penile urethra attached to the corpora, and glans volume. We measured the full volume of the glans before and after resection of the dorsal glandial ridge that was used for the reconstruction of the new clitoris. The volume of the glans was measured by using circumference and height, according to the cone volume formula (V = 1/3πr^2^h). We calculated the total and remaining glans volume and calculated the precise remnant volume that would be used for tissue transplantation together with the fully preserved corpora cavernosa and anterior urethra. Additionally, we evaluated the presence of the neurovascular elements, deep dorsal vein, penile arteries, penile nerves, crural arteries, and veins as well all other elements such as perforant and circumflex branches and nerve endings. No statistical analysis, except standard deviation, was performed since the study was not comparative.

## 3. Results

The length of the corpora cavernosa after removal ranged from 17.7 to 24.6 cm (mean 20.46 cm, SD 1.74). Girth was measured at the median level, ranging from 5.7 to 11.2 cm (mean 8.45 cm, SD 1.53). Glans volume was measured before and after the excision of the glans ridges that were used for the new clitoris. The mean glans volume before and after the glans excision was 5.12 mL, SD 0.76 (3.89–6.44 mL) and 4.38 mL, SD 0.672 (3.27–5.56 mL), respectively. The remnant glans volume was between 83.9 and 86.9% of the total volume (mean 85.37%). The remaining urethra was measured from the urethral opening at the glans to the dividing lane and ranged from 12.7 to 16.4 cm (mean 13.94 cm, SD 0.99) (Table 1). Microvascular dissection of the neurovascular bundle enabled precise identification of all elements, the deep dorsal vein, the two penile arteries, and the two penile nerves. The distribution of the neurovascular bundle was determined, and regular anatomy was found in thirty-six cases, while in the remaining five (12.2%), one penile artery was completely missing. The crural arteries were identified in all cases and ligated at the level of bifurcation with the bulbourethral arteries. The penile nerves and deep dorsal veins were found in all cases with the usual anatomical distribution. The penile and scrotal skin were preserved in all cases and were sufficient for neovaginal reconstruction. The length of the penile urethra was sufficient in all cases and adequate for joining with the female urethra of the potential recipient.

The mean (range) follow-up time was 15 months (from 6 to 26 months). Good depth of the neovagina was achieved in all patients and ranged from 11.5 to 14 cm, 12 to 14.8 cm, and 13 to 18 cm in the penile inversion, peritoneal flaps, and sigmoid segments, respectively. It was estimated in the present patients using vaginal dilators. The patients’ satisfaction with the moisture of the neovagina, sensation, and orgasms were obtained by interviewing the patients, and were satisfactory in 29, 41, and 31 patients, respectively. The genitalia had an aesthetically pleasant appearance in thirty-seven patients, while in the remaining four, minor corrections were necessary.

## 4. Discussion

Feminizing gender-affirming surgery represents the last step in transfemale transitions. The set of surgical procedures are aimed at aligning an individual’s physical appearance with their gender identity. For transgender women, these surgical procedures typically involve feminization of the chest, face, and genitalia [17]. The main goal is to create esthetically and functionally acceptable female genitalia after testicle and penis removal. The penis removal is based on the separation of the corpora cavernosa from a small part of the glans with the neurovascular bundle and from the proximal urethra that will be used for the reconstruction of the new clitoris and female urethral orifice. The dorsal segment of the glans supported with nerve and blood elements is reshaped, making a sensitive clitoris. Additionally, the urethra is mobilized and spatulated, and the bulbar part is used for the creation of the female urethral orifice while the distal part is joined with the glans clitoris, creating the vulvo-vestibular complex [18]. Our experience of more than 30 years in this field raised ideas on how to use removed remnants as possible tissue for live-donor penile transplantation [14,19,20].

Even though neophalloplasty is the most used penile replacement method, the success rate of these procedures and the patients’ satisfaction are limited [21]. Optimal phalloplasty should offer the following conditions: good volume and sensitivity of the neophallus, possibility for insertion of erectile devices for penetrative sexual intercourse, creation of a long enough neourethra with the possibility for voiding while standing, minimal stages, and acceptable scar formations. Some of the complications include tissue necrosis and urethral complications, such as strictures or fistulas, blood clots, and deep vein thrombosis. Also, depending on the technique of choice, neophalloplasty can result in altered or diminished sensation in the neophallus and surrounding areas. Furthermore, these complications, poor cosmetic results, and non-satisfactory erectile function remain and present a great challenge for better options [2,3,22]. The vascularized composite allotransplantation became an option for the treatment of complex hand and face defects, making it a feasible alternative for genital reconstruction [23,24]. The first experiences in penile transplantation from cadavers are promising, and all details remain unknown [25]. The possibility of using vascularized penile tissues from live donors i.e., transwomen, makes our ideas more promising. As the number of transgender surgeries continues to increase globally, in candidates who have requested feminizing gender affirmation procedures, the removed penile tissue (corpora cavernosa, remaining volume of the glans and anterior urethra) could be potentially suitable for live-donor transplantation [26].

In the last two decades, five penile transplantations were reported from four different centers with versatile outcomes. The first was performed in China in 2006, but the penis was explanted two weeks later due to psychological instability from both the patient and his wife [4,5]. Two cases were reported from Cape Town in 2014 and 2017, and the first one remains attached to date while another was explanted in 2021 due to several episodes of rejection and tissue necrosis [6,7]. The remaining two cases, from Baltimore and Boston, were performed in 2016 and 2018, respectively [8,9]. Recent data confirmed a stable situation with very good functional outcomes [25]. The Baltimore criteria for an ethical approach to penile transplant were established to appropriately distinguish suitable candidates, major postoperative concerns, as well as ethical and privacy dilemmas [27]. Except for patients that have lost their penis due to trauma or cancer, the Baltimore criteria also include patients who would not accept other options for penile reconstruction, and in this context, transgender patients can be sorted into that group. Another presenting problem with cadaveric transplantation would be the willingness of the donor’s families to consent to such a form of organ donation, considering that people are still uneducated about transgender people, their transition, and their need for gender-affirming surgery.

In all reported cases, the allograft was taken from a suitable cadaveric donor, with a cold ischemia time of minimum 16 h. Even though transplant surgeons have practiced the transplantation techniques, microsurgical reconstruction, and cadaver-to-cadaver transplantation, some of the questions remain [25,27]. Due to the experimental nature of the procedure, there are potential risks and challenges, including the risk of rejection, the need for lifelong immunosuppressive medications, and psychological implications for both the donor’s family and the recipient. The allograft harvesting in the cadavers usually lasts longer due to other multi-organ donors, resulting in longer cold ischemia. On the other hand, live-donor tissue harvesting represents the ideal solution for transplantation but only for selected organs such as the kidneys, liver segments, bone marrow, and skin. Based on our experience in penile tissue removal during gender-affirming vaginoplasties, we developed a very precise microdissection of all elements of the penis, preparing the corpora cavernosa with a good volume of the glans and with the distal urethra as an excellent tissue for possible live-donor transplantation.

This is a unique research study, based on penile reconstructive surgery and separated into several steps. According to the literature, appropriate animal models are limited and just few of the available reports described possible transplantation in rats and dogs with good success rates [28,29,30]. In our patients receiving gender-affirming vaginoplasties, we performed precise microvascular dissections of the penis, based on penile disassembly principles, and preserved all penile tissue that is normally removed during reconstruction. This material is feasible to be used for potential live-donor penile transplantation. The next step will be to develop techniques for tissue harvesting, transfer, and microvascular transplantation.

Our approach for microdissection is based on a penile disassembly technique that enables precise separation of the corpora cavernosa from all other parts of the penis such as the urethra and the glans cap with the neurovascular bundle. We previously reported penile disassembly in the treatment of very severe penile deformities, preserving all penile structures with their functional assembly after correcting the anomaly [15,16]. We used this principle for the dissection of the corpora cavernosa from the neurovascular bundle dorsally and the bulbar urethra ventrally. This way, the anterior urethra and glans remnant remain firmly attached to the corpora cavernosa, enabling a good vascular supply from the corpora. This fact could be very important for the survival of these structures after potentially joining with the recipient.

Our study provides some interesting key points for those who would like to start a penile transplantation program. The main purpose was to offer methodological guidance for the way in which micro anatomical dissection of the penis may play a role in the preparation of the penile tissue for possible penile transplantation. The method of using dorsal neurovascular elements as a part of the reconstruction of the neoclitoris presents a great challenge in attempts to preserve the blood supply of the glans remnant and anterior urethra. In our study, the presence of one dorsal penile artery was found in five cases (12.2%). In all of them, the newly formed clitoris survived, and excellent sensation was reported. Since the clitoris in these patients was supported by the one dorsal penile artery, the deep dorsal vein, and one penile nerve, we hypothesized that it will be enough, and another artery with one dorsal nerve could be preserved supporting the survival of the glans remnant. In this way, the remaining artery and nerve could be sufficient for glans remnant survival after transplantation. Furthermore, venous drainage of the glans is mainly based on the deep dorsal vein and its branches. We theorized that the distal part of the urethra, which is connected to the glans and anterior part of the corpora cavernosa, enables additional venous drainage from the glans. Our dissection of the urethra is performed just at the bulbar level since the short segment of the anterior urethra is usually necessary for the creation of the new female orifice. Almost the entire anterior urethra, very rich in blood vessels due to its connection to the tunica albuginea, could be preserved and used for joining with the original urethra in the recipient, giving additional blood supply to other structures, corpora, and glans. Joining with the proximal part of the urethra in penile amputation could be very easy and safe. The blood supply from the proximal urethra could be enough to fill and empty preserved spongiosal tissue of the anterior urethra. The same situation is visible in transmen, and the joining of a donor anterior urethra with a good vascularized female urethra makes this method more realistic. Experience in urethral reconstructive surgery supports these facts. Excision and primary anastomotic urethroplasty (EPA) presents the method of choice for the treatment of the most strictures of the bulbar urethra. The technique is based on excision of the scar and extensive mobilization of both the proximal and distal urethral parts for direct anastomosis without tension [31]. We avoid additional maneuvers such as anterior urethral dissection and mobilization from the corpora cavernosa to preserve the corporal blood flow to the urethra. The male urethra has been shown to be exceptionally extensible, with a possible additional 65% of length obtained after mobilization, allowing for a tension-free anastomosis even in longer distance [32]. Mobilization of the proximal urethral bulb in the recipient, either in a man or transman, presents a key of success for additional length that is needed for safe anastomosis to the donor anterior urethra without tension and injury of the dorsal perforating vasculature.

Penile structures were measured during dissection to estimate the quality and quantity of the tissue after using the parts necessary for the reconstruction of the female genitalia. These results showed an excellent potential of penile tissue remnants, such as the length of the corpora and urethra and the volume of the remaining glans tissue. All remaining parts of the penis remained attached without separation and with preserved blood vessels between entities. The length of the corpora cavernosa was completely preserved with an average of 20.46 cm which could be sufficient for microvascular transfer to a potential recipient. The glans volume after dissection was between 83.9 and 86.9% of the total volume, presenting a viable tissue for transplantation. Finally, the mean length of the resected urethra (13.94 cm) enables easy and safe joining with the recipient’s urethra. According to bioethical aspects, good female genitalia were created as a primary goal in all cases, while microdissection and remnant removal did not jeopardize the outcome of gender-affirming vaginoplasty. Contrary to reported cases of penile transplantation, we found a lack of available donor penile skin that is usually used for standard gender-affirming vaginoplasty, leaving denuded corpora cavernosa as an unsuitable tissue for transplantation [18,27]. We do not consider that as a disadvantage. The lack of skin could be solved either by the recipient’s genital skin flaps or by auto-transplantation of the split-thickness skin grafts. The latter could be a better solution since the allotransplantation of the skin presents a high risk for a quick immune rejection process [33]. It is well-known that the skin has extreme antigenic characteristics and induces very intensive immune responses after allotransplantation.

The possibility for live-donor penile transplantation still exists. Besides surgical aspects, other medical and ethical issues remain. The purpose of our study was to investigate the anatomical and technical feasibility of the surgical procedures for live-donor penile transplantation. This research confirmed the safe and precise microdissection of penile entities. Based on ethical principles, we used all available penile tissue for the reconstruction of female genitalia as previously described. The main lack of our study is the absence of a model for transplantation that should incorporate all anastomosis, urethral joining, and nerve connection to the recipient body. Further research is necessary, and it is going to be a new part of our project.

## 5. Conclusions

Feminizing gender affirmation surgery was precisely described with all anatomical principles. Microdissection of the penis was defined as a safe and simple procedure with detailed harvesting and preservation of the remaining penile entities after the creation of new female genitalia. This way, preserved corpora cavernosa with anterior urethra ventrally, and good volume of the glans could be used for safe and successful live donor penile transplantation. However, our preliminary results should be confirmed by further research with the aim to improve the technical possibilities and offer standardization of operative techniques, providing the ideal male genital organ in all aspects.

## Figures and Tables

**Table 1:** Measurement of penile entities during penile microdissection.

No.	Age	Type of Surgery	Corporal Length (cm)	Corporal Girth (cm)	Urethral Length (cm)	Total Glans Volume (mL)	Glans Remnant (mL)	Glans Remnant Volume (%)
1.	26	SV	20.1	8.1	13.5	5.00	4.26	85.2
2.	35	PIV	21.5	10.0	14.6	5.66	4.81	85
3.	43	PPV	22.5	10.8	15.2	6.13	5.21	85
4.	28	PIV	20.7	8.9	13.8	5.23	4.45	85
5.	20	PIV	18.2	6.5	12.8	4.04	3.39	84
6.	20	PPV	18.4	7.1	12.9	4.16	3.54	85
7.	22	SV	18.6	7.2	13.3	4.34	3.69	85
8.	36	PPV	22.8	8.5	15.2	6.20	5.33	86
9.	28	PPV	20.5	8.4	13.8	5.20	4.48	86
10.	20	PIV	17.9	6.4	12.8	3.96	3.39	85.6
11.	51	PIV	23.4	10.2	15.9	6.33	5.50	86.8
12.	47	PPV	23.1	8.8	15.6	6.31	5.42	85.9
13.	21	PPV	22.2	10.4	14.9	6.08	5.17	85
14.	20	PPV	22.0	9.4	14.8	6.00	5.15	85.8
15.	32	PIV	20.9	10.5	14.4	5.56	4.72	84.9
16.	29	PIV	20.8	9.0	14.1	5.31	4.51	84.9
17.	35	PPV	21.4	9.5	14.5	5.63	4.78	84.9
18.	31	PIV	20.8	10.3	14.2	5.47	4.65	85
19.	29	PIV	19.5	7.6	13.2	4.71	4.05	85.9
20.	37	SV	21.7	11.0	14.7	5.77	4.92	85.2
21.	22	PIV	18.8	7.5	13.3	4.35	3.69	84.7
22.	59	PIV	20.1	7.8	13.5	4.92	4.23	86
23.	26	PIV	20.4	8.0	13.2	5.06	4.25	84
24.	22	PIV	19.1	7.5	13.5	4.57	3.88	84.9
25.	41	PIV	18.5	6.9	12.9	4.22	3.67	87
26.	20	PIV	23.7	10.7	15.9	6.33	5.50	86.8
27.	31	PPV	20.2	6.6	13.0	4.63	4.03	87
28.	29	PPV	19.4	7.7	13.1	4.66	4.07	87.3
29.	28	PIV	19.8	7.7	13.2	4.77	4.05	84.9
30.	22	PPV	18.9	6.8	13.4	4.48	3.76	83.9
31.	34	PIV	21.4	10.0	14.5	5.63	4.78	84.9
32.	37	PPV	21.8	8.2	14.7	5.77	4.92	85.2
33.	54	PPV	19.2	7.0	13.0	4.60	3.91	85
34.	26	PIV	20.3	8.0	13.5	5.10	4.38	85.8
35.	32	PIV	21.2	9.7	14.4	5.59	4.75	85
36.	26	SV	18.6	6.7	13.0	4.29	3.64	84.8
37.	27	PIV	24.6	11.2	16.4	6.40	5.56	86.9
38.	32	PPV	17.9	6.2	12.8	3.92	3.33	84.9
38.	25	PIV	19.8	7.9	13.4	4.84	4.12	85.1
40.	19	PIV	17.7	5.7	12.7	3.89	3.27	84.1
41.	44	PPV	23.0	10.4	15.2	6.20	5.33	86
MEAN	30.5	-	20.46	8.45	13.94	5.12	4.38	85.37
SD	-	-	1.74	1.53	0.99	0.76	0.67	0.85

PIV—penile inversion vaginoplasty; SV—sigmoid colon vaginoplasty; PPV—peritoneal pull-through vaginoplasty; SD—standard deviation.

## Data Availability

Data are contained within the article.

## References

[1] McNichols C.H.L., O’Brien-Coon D., Fischer B. (2020). Patient-reported satisfaction and quality of life after trans male gender affirming surgery. Int. J. Transgend. Health.

[2] Robinson I.S., Blasdel G., Cohen O., Zhao L.C., Bluebond-Langner R. (2021). Surgical Outcomes Following Gender Affirming Penile Reconstruction: Patient-Reported Outcomes from a Multi-Center, International Survey of 129 Transmasculine Patients. J. Sex. Med..

[3] Garaffa G., Ralph D.J. (2016). Free Flap Phalloplasty for Female to Male Gender Dysphoria. J. Sex. Med..

[4] Hu W., Lu J., Zhang L., Wu W., Nie H., Zhu Y., Deng Z., Zhao Y., Sheng W., Chao Q. (2006). A preliminary report of penile transplantation. Eur. Urol..

[5] Hu W., Lu J., Zhang L., Wu W., Nie H., Zhu Y., Deng Z., Zhao Y., Sheng W., Chao Q. (2006). A preliminary report of penile transplantation: Part 2. Eur. Urol..

[6] Van der Merwe A., Graewe F., Zühlke A., Barsdorf N.W., Zarrabi A.D., Viljoen J.T., Ackermann H., Spies P.V., Opondo D., Al-Qaoud T. (2017). Penile allotransplantation for penis amputation following ritual circumcision: A case report with 24 months of follow-up. Lancet.

[7] van der Merwe A., Toefy Y., Moosa M.R., van Deventer H., Scott C.J. (2021). Living with someone else’s penis: The lived experiences of two South African penile allograft recipients: A descriptive phenomenological study. Ann. Med. Surg..

[8] Cetrulo C.L., Li K., Salinas H.M., Treiser M.D., Schol I., Barrisford G.W., McGovern F.J., Feldman A.S., Grant M.T., Tanrikut C. (2018). Penis transplantation: First US experience. Ann. Surg..

[9] Redett R.J., Etra J.W., Brandacher G., Burnett A.L., Tuffaha S.H., Sacks J.M., Shores J.T., Bivalacqua T.J., Bonawitz S., Cooney C.M. (2019). Total penis, scrotum, and lower abdominal wall transplantation. N. Engl. J. Med..

[10] Dunford C., Bell K., Rashid T. (2021). Genital Reconstructive Surgery in Male to Female Transgender Patients: A Systematic Review of Primary Surgical Techniques, Complication Profiles, and Functional Outcomes from 1950 to Present Day. Eur. Urol. Focus.

[11] van der Sluis W.B., Schäfer T., Nijhuis T.H.J., Bouman M.B. (2023). Genital gender-affirming surgery for transgender women. Best. Pract. Res. Clin. Obstet. Gynaecol..

[12] O’Dwyer C., Kumar S., Wassersug R., Khorrami A., Mukherjee S., Mankowski P., Genoway K., Kavanagh A.G. (2023). Vaginal self-lubrication following peritoneal, penile inversion, and colonic gender-affirming vaginoplasty: A physiologic, anatomic, and histologic review. Sex. Med. Rev..

[13] Morrison S.D., Claes K., Morris M.P., Monstrey S., Hoebeke P., Buncamper M. (2023). Principles and outcomes of gender-affirming vaginoplasty. Nat. Rev. Urol..

[14] Perovic S., Stanojevic D., Djordjevic M. (2000). Vaginoplasty in male transsexuals using penile skin and a urethral flap. Br. J. Urol..

[15] Perovic S., Vukadinovic V., Djordjevic M., Djakovic N. (1998). The penile disassembly technique in hypospadias repair. Br. J. Urol..

[16] Perovic S., Djordjevic M. (2001). The penile disassembly technique in the surgical treatment of Peyronie’s disease. BJU Int..

[17] Anderson D., Wijetunge H., Moore P., Provenzano D., Li N., Hasoon J., Viswanath O., Kaye A.D., Urits I. (2022). Gender Dysphoria and Its Non-Surgical and Surgical Treatments. Health Psychol. Res..

[18] Li V.Y., Demzik A., Snyder L., Ogunleye A.A., Wang A., Figler B.D. (2022). Genital Gender Affirming Surgery. Am. Surg..

[19] Stanojevic D.S., Djordjevic M.L., Milosevic A., Sansalone S., Slavkovic Z., Ducic S., Vujovic S., Perovic S.V., Team B.G.D. (2007). Sacrospinous ligament fixation for neovaginal prolapse prevention in male-to-female surgery. Urology.

[20] Bizic M.R., Stojanovic B., Djordjevic M.L. (2017). Genital reconstruction for the transgendered individual. J. Pediatr. Urol..

[21] Özer M., Toulabi S.P., Fisher A.D., t’Sjoen G., Buncamper M.E., Monstrey S., Bizic M.R., Djordjevic M., Falcone M., Christopher N.A. (2022). ESSM Position Statement "Sexual Wellbeing After Gender Affirming Surgery". Sex. Med..

[22] Pang K.H., Christopher N., Ralph D.J., Lee W.G. (2023). Insertion of inflatable penile prosthesis in the neophallus of assigned female at birth individuals: A systematic review of surgical techniques, complications and outcomes. Ther. Adv. Urol..

[23] Khalifian S., Brazio P.S., Mohan R., Shaffer C., Brandacher G., Barth R.N., Rodriguez E.D. (2014). Facial transplantation: The first 9 years. Lancet.

[24] Shores J.T., Brandacher G., Lee W.P.A. (2015). Hand and upper extremity transplantation: An update of outcomes in the worldwide experience. Plast. Reconstr. Surg..

[25] Lake I.V., Girard A.O., Lopez C.D., Cooney D.S., Burnett A.L., Brandacher G., Oh B.C., Redett R.J. (2022). Penile Transplantation: Lessons Learned and Technical Considerations. J. Urol..

[26] Stojanovic B., Radnic B., Bizic M., Bogdanovic M. (2023). Cadaveric penile dissection and its impact on live donor penile transplantation. Eur. Urol. Suppl..

[27] Ngaage L.M., Elegbede A., Sugarman J., Nam A.J., Cooney C.M., Cooney D.S., Rasko Y.M., Brandacher G., Redett R.J. (2020). The Baltimore Criteria for an ethical approach to penile transplantation: A clinical guideline. Transpl. Int..

[28] Koga H., Yamataka A., Wang K., Kato Y., Lane G.J., Kobayashi H., Sueyoshi N., Miyano T. (2003). Experimental allogenic penile transplantation. J. Pediatr. Surg..

[29] Seyam R.M., Kattan S.A., Assad L.W., El-Sayed R.M., Almohanna F.H. (2013). Penile autotransplantation in rats: An animal model. Urol. Ann..

[30] Zhao Y., Hu W., Zhang L., Guo F., Wang W., Wang B., Zhang C. (2016). Penis Allotransplantation in Beagle Dog. Biomed. Res. Int..

[31] Morey A.F., Watkin N., Shenfeld O., Eltahawy E., Giudice C. (2014). SIU/ICUD Consultation in Urethral Strictures: Anterior urethra-primary anastomosis. Urology.

[32] Erickson B.A., Granieri M.A., Meeks J.J., Cashy J.P., Gonzalez C.M. (2010). Prospective analysis of erectile dysfunction after anterior urethroplasty: Incidence and recovery of function. J. Urol..

[33] Richters C.D., Hoekstra M.J., Du Pont J.S., Kreis R.W., Kamperdijk E.W.A. (2005). Immunology of skin transplantation. Clin. Dermatol..

